# Direct and indirect effects of self-directed learning on creativity in healthcare undergraduates: a chain mediation model of openness to challenge and diversity and creative self-efficacy

**DOI:** 10.3389/fpsyg.2023.1182692

**Published:** 2023-06-12

**Authors:** Jiazhen Qian, Xiang Li, Ting Liu, Mengfan Zhang, Kaiyan Li

**Affiliations:** School of Nursing, Qingdao University, Qingdao, China

**Keywords:** creativity, self-directed learning, openness to diversity and challenge, creative self-efficacy, healthcare undergraduates

## Abstract

**Background:**

Creativity and self-directed learning (SDL) are prominent for undergraduate healthcare students to provide quality patient care in an increasingly complex healthcare environment. Research suggested that SDL is linked with creativity, yet the mechanism underlying the relationship between SDL and creativity has not been fully understood.

**Objective:**

This study examined the relationship between SDL and creativity and constructed a chain mediation model to identify the mediating effect of openness to diversity and challenge (ODC) and creative self-efficacy (CSE).

**Methods:**

Through convenience sampling, 575 healthcare undergraduates (average age = 19.28 years, *SD* = 1.124 years) were surveyed from Shandong Province in China. Creativity, SDL, ODC, and CSE were assessed using corresponding scales. Pearson’s correlation analysis, hierarchical multiple linear regression analysis, a serial multiple mediation analysis, and bias-corrected percentile Bootstrap method were conducted by using structural equation modeling by AMOS 26.0.

**Results:**

The direct path between SDL and creativity was significant. SDL can positively predict both ODC and CSE, and the latter two variables can significantly and positively predict creativity. ODC and CSE played a significant partial mediating role in the relationship between SDL and creativity. The mediating effect consists of three indirect effects: SDL → ODC → creativity (the mediating effect value is 0.193, *p* = 0.012), SDL → CSE → creativity (the mediating effect value is 0.096，*p* = 0.001), and SDL → ODC → CSE → creativity (the mediating effect value is 0.035, *p* = 0.031).

**Conclusion:**

SDL can positively predict creativity. ODC and CSE had significant mediating effects between SDL and creativity, including single partial mediating effects of ODC and CSE and chain mediating effects of ODC-CSE.

## 1. Introduction

Creativity is acknowledged as an essential component of individuals’ ability to deal with contemporary social challenges (Liu et al., 2019). Creativity is the ability to transcend traditional ideas, patterns, rules or relationships and to generate meaningful new concepts, forms, methods, interpretations and solutions to problems (Runco and Jaeger, 2012; Koh, 2013). The healthcare landscape is altering rapidly owing to the acceleration of aging populations, complexities of disease management, and advancement of health technologies. To effectively respond to the approaching challenges and sustain high-quality care, healthcare students need to be prepared with capabilities for creativity to identify and figure out problems in their learning and working environments (Liu et al., 2019, 2021). In China, medical education is experiencing changes and challenges especially in the context of maintenance of people’s health being the national strategy (The State Council of China, 2016). For instance, graduates with bachelor’s degrees or above occupied three quarters of the higher clinical medical graduates from 2015 to 2018 in China, in which junior medical colleges cultivate some graduates for primary health care that mainly train nurse and allied health professionals (Liu et al., 2023). Simultaneously, educational institutions place great importance to the cultivation of healthcare students’ health professionalism and innovation. Nonetheless, medical schools in China seem to have a slow response to external demands, in which the pedagogic methods are mainly teacher-controlled didactic lecturing in the classroom (Wang, 2021). These methods are perceived as potentially inhibiting students’ creativity (Yang et al., 2018). Accordingly, scholars advocate cultivating creative thinking of healthcare students as one of the directions to improving the competence of graduates who will contribute to high quality healthcare (Koh, 2013; Wang, 2021).

Regarding creativity, people hold an implicit theory regarding its fixed or malleable feature (i.e., growth mindset) (Karwowski et al., 2018). A growth mindset is the belief that human capacities are not fixed but can be developed over time (Dweck and Yeager, 2019). Accordingly, the belief concerning the malleable nature of creativity is known as “creative mindsets.” Previous research revealed that creative growth mindset positively predicted interest in creative thinking and creative performance (Intasao and Hao, 2018). With the growth mindset, creativity can be fostered individually and organizationally through education or training at school and the workplace (Miller, 2008). Specifically, the Dual Pathway to Creativity Model asserts that creativity can be achieved by two cognitive pathways, i.e., flexibility pathway and the persistence pathway (Bernard et al., 2010; Baas et al., 2013). In other words, creativity is obtained through cognitive flexibility that is manifested in divergent thinking with characteristics of generation of many varied and original options. For the cognitive persistence pathway, individuals draw many ideals from a few categories, that is called within-category fluency (Roskes et al., 2012). Although both cognitive pathways contribute to creativity, the flexibility pathway is regarded to be more effective compared to the persistence pathway because the latter requires more cognitive resources (Roskes et al., 2012) and divergent thinking has long been regarded as the key ability underlying creative performance (Mumford and England, 2022).

### 1.1. Theoretical framework and research hypotheses

#### 1.1.1. Self-directed learning and creativity

In educational field, research found that self-directed learning directly influenced the problem-solving ability that is associated with critical thinking (Song et al., 2022). In the meantime, critical thinking was found to be positively linked to creative thinking (Liu et al., 2021). Therefore, self-directed learning may be related to creativity. Self-directed learning (SDL) is identified as critical in diverse educational settings and is an essential feature for lifelong learning (Taylor and Hamdy, 2013). Knowles (1975, p. 18) described SDL “as a process in which individuals take initiative, with or without the help of others, in diagnosing their learning needs, formulating learning goals, identifying human and material resources for learning, choosing and implementing appropriate learning strategies, and evaluating learning outcomes.” This process-based definition aligns with self-determination theory that claims three innate human psychological needs (i.e., autonomy, competency, and relatedness) determine the ongoing psychological growth toward integrity and well-being (Ryan and Deci, 2000). According to self-determination theory, human beings are naturally inclined to develop autonomous regulation of behavior and are intrinsically motivated to learn and to take on challenges (Ten Cate et al., 2011). SDL is commonly defined as the ability and attitude of students to develop and pursue their own learning objectives and to evaluate their learning process and results (Abd-El-Fattah, 2010; van Woezik et al., 2021). Future healthcare professionals require SDL in highly dynamic and diversified settings in which learners assume the principal responsibility for their own learning (Murad et al., 2010; van Woezik et al., 2021). The importance of SDL for uncertain times such as the COVID-19 pandemic has attracted attention in the training of future healthcare professionals (Singaram et al., 2022).

SDL was found to be positively correlated with various learning outcomes including enhanced confidence, intrinsic motivation to learn, critical thinking, and creativity (Lunyk-Child et al., 2001; Zhoc et al., 2018; Shafait et al., 2021). Previous studies in various adult learning contexts suggested a relationship between SDL and creativity. A systemic analysis of vocational education of young adult learners in England found that creative learning outcomes were evident when students played a role of directing the learning process rather than the teachers dominating the control over the learning process (Morris, 2018). Through a quasi-experimental design, Yang et al. (2018) demonstrated that challenge-based learning with a component of self-directed learning could enhance undergraduate nursing students’ ability to innovate and create. A study on higher vocational students demonstrated that when students consider how to solve problems, they would be inspired with more creativity and creative ideas (Li et al., 2022). This learner autonomy and independence is in keeping with the key propositions of self-determination theory that highly self-directed learners are intrinsically motivated to learn. Informed by self-determination theory and related studies, the following hypothesis was proposed.

*H1:* Self-directed learning has a positive effect on creativity among healthcare undergraduates.

#### 1.1.2. The mediating role of openness to diversity and challenge

Openness has long been recognized as an important personality of creativity as it help individuals to consider different viewpoints that serve as an essential prerequisite for creativity (Tidikis and Dunbar, 2019). Openness to experience personality is related to different modes of information processing and predict creative achievement in arts and sciences (Tidikis and Dunbar, 2019). Openness to experience is related to openness to diversity and challenge, Bowman (2014) pinpointed that the two variables were different because openness to diversity and challenge (ODC) was a state that can be influenced by situational factors (e.g., university experience) while openness to experience represented a relatively stable trait. ODC is defined as individuals’ willingness to change their own beliefs and values, and to interact and learn from others who are different from themselves, that reflects a preference for novelty and difference over the conventional (Whitt et al., 2001). ODC is considered as a critical disposition for flourishing within an increasingly diverse and globalized society for college students (Bowman, 2014). In higher education environments, ODC advances students to engage in meaningful educational experiences and quality peer interactions (Bowman, 2014), and stimulates exploration of previously unknown ideas (van Woezik et al., 2021). ODC was positively related with creative confidence beliefs in higher education students in Spain (Álvarez-Huerta et al., 2022). In this sense, ODC may play a role in creativity among healthcare undergraduates yet has been less explored, especially in Chinese context.

In addition, openness to experience was also found to be the most characteristic personality trait of self-directed learners (Cazan and Schiopca, 2014). Personal attributes are the key elements of SDL that encompass curiosity, perseverance, flexibility, and adaptability (Ricotta et al., 2022). For instance, a flexible learner embraces new ideas, and appreciates new perspectives, and has the ability to adapt creatively to new challenges. These attributes theoretically connect to ODC. An study on first-year undergraduates found that self-directed learning had significant association with cognitive outcomes (e.g., dealing with unfamiliar problems, viewing things from a global perspective) and social learning outcomes (e.g., getting along with people of different cultural and ethnic backgrounds) (Zhoc et al., 2018). These outcomes echo with the ODC, and thus we speculate SDL could promote ODC. Based on the associations among SDL, creativity and ODC, the following tentative hypothesis was developed.

*H2:* Self-directed learning significantly and positively predicts creativity among healthcare undergraduates through the mediating effect of ODC.

#### 1.1.3. The mediating role of creativity self-efficacy

According to Social Cognitive Theory (SCT), self-efficacy is a key motivational process that results in outcomes including choices of activities, effort, persistence and achievement (Schunk and DiBenedetto, 2021). In other words, individuals with higher self-efficacy are inclined to choose to engage in activities, expend greater efforts, persist longer and achieve at higher levels compared with those who feel less efficacious. Creative self-efficacy (CSE) refers to individuals’ confidence in their ability to execute and fulfill the specific tasks that pertain to creativity or innovation (Tierney and Farmer, 2002). Creative self-efficacy is viewed as an antecedent to creativity as it determines the extent of individuals’ attempt to undertake creative tasks, the intensity of efforts, and the persistency in the face of difficulty (Tierney and Farmer, 2002). Individuals with high levels of CSE could have sufficient positive psychological capital to handle uncertainties and difficulties, and thus tend to mobilize motivation, cognitive resources, and actions to meet contextual demands (Qiang et al., 2020). By contrast, those holding a creative potential may withdraw from challenging situations due to low CSE (Rogaten and Moneta, 2016).

Being the perceived confidence that individuals have regarding their knowledge and ability to generate new and adaptive ideas, solutions, and creative things, CSE is positively linked with creative performance and creative mindset (Royston and Reiter-Palmon, 2017; Liu et al., 2021). Empirical research confirmed that creative self-efficacy played a partially mediating role in the association between transformational tutoring style and innovation behaviors among postgraduate students (Ma et al., 2023). For scientific research teams in Chinese higher educational settings, creative self-efficacy mediated the relationship between benevolent leadership and team creative performance (Xia et al., 2021). Creative self-efficacy could stimulate intrinsic motivation to pursue innovation, and thus is closely connected with creativity.

There are four key sources of CSE (i.e., mastery experiences, vicarious experiences, verbal persuasion, and physiological/affective states) that individuals use to assess their self-efficacy (Puente-Diaz, 2016). Past experiences shape people’s current beliefs that drive their future actions (Intasao and Hao, 2018). Through the self-directed learning process, individuals’ need of autonomy, competence and relatedness are met (Ten Cate et al., 2011), which link with the four sources of CSE and thus boost their confidence in creativity. Likewise, Ma et al. (2023) asserted that enhancing students’ own concentration and commitment and encouraging them to actively undertake challenging tasks are conducive to creative self-efficacy. With the above analysis, hypothesis H3 was formulated based on Social Cognitive Theory.

*H3:* Self-directed learning significantly and positively predicts creativity among healthcare undergraduates through the mediating effect of CSE.

#### 1.1.4. The chain mediating roles of openness to diversity and challenge and creative self-efficacy

A recent study revealed ODC as a mediator in the relationship between critical thinking disposition and creative confidence beliefs among college students in Spain (Álvarez-Huerta et al., 2022). Students who were more open to diversity and challenge had a stronger creative self-concept that acts as a crucial factor underpinning creative behavior and creative outcomes (Lebuda et al., 2020). In addition, for employees, ODC was reported to have a direct positive relationship with creative self-efficacy and an indirect positive relationship with creativity via creative self-efficacy (Gong et al., 2019). Openness to experience as a team personality component reinforced the relationship between creative self-efficacy and team creative performance in higher educational institutions (Xia et al., 2021). Puente-Diaz (2016) proposed that openness to experience appeared to be a positive predictor of creative self-efficacy. In this sense, besides connection with creativity, ODC may contribute to creative self-efficacy. As a result, H4 was assumed.

*H4:* Openness to diversity and challenge and creative self-efficacy play a chain mediating role between self-directed learning and creativity among healthcare undergraduates.

**Figure 1:**
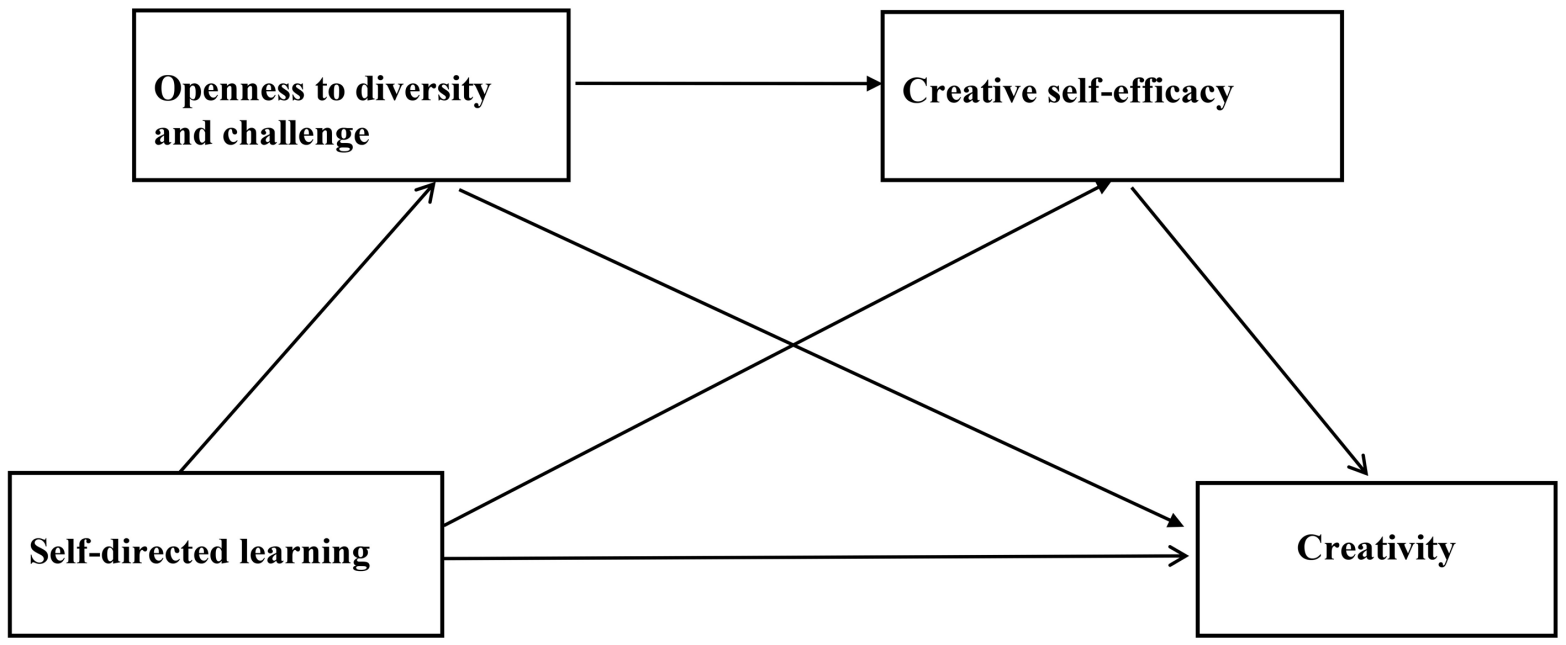
The proposed mediation model.

Taken together, literature review indicated that there may be complicated relationships among SDL, ODC, CSE and creativity, yet the mechanism underlying the relationship between SDL and creativity remains unclear. The present study aimed to explore the mechanism of the relationship between SDL and creativity of healthcare undergraduates to verify the four tentative hypotheses described above. A serial mediation model (Figure 1) was proposed to test the mediating roles of ODC and CSE in the association between SDL and creativity.

## 2. Materials and methods

### 2.1. Participants and procedure

A descriptive quantitative research design was adopted by using self-administered survey to investigate creativity, SDL, ODC, CSE and explore their relationships. Convenient sampling was utilized to recruit participants from the Faculty of Medicine at a public and comprehensive university in Shandong Province of China. The inclusion criteria were (1) aged ≥18 years; (2) being a full-time undergraduate; (3) enrolled in a healthcare program (e.g., clinical medicine, dentistry, preventive medicine, nursing, pharmacy, etc.); and (4) willing to participate in the study. For multiple mediator model, research suggested that 500 sample size was required to detect significant mediating effect if the indirect effect was small (Ma, 2014). A total of 626 questionnaires were distributed and 575 valid questionnaires were returned, generating an effective response rate of 91.85%.

This study was approved by the Ethics Committee of Medical College of Qingdao University where the study was conducted (QDU-HEC-2022044). The investigators explained the purpose of the study, voluntary participation, nonmaleficence, data confidentiality and how to withdraw from the survey to the potential participants. Participants provided informed consent prior to participation in the study. The survey took 10–15 min to complete.

### 2.2. Questionnaires

#### 2.2.1. General information questionnaire

Based on related research on creativity (Liu et al., 2021; Beaulieu, 2022), the research team designed the sociodemographic questionnaire, including age, gender, grade, academic performance, residence, being the only child or not in the family, and parents’ education level.

#### 2.2.2. The university students’ creativity scale

The University Students’ Creativity Scale was used to measure healthcare undergraduates’ creativity. The scale was developed by Chinese scholars (He et al., 2015) based on Sternberg’s implicit theory of intelligence, creativity and wisdom (Sternberg, 1985). The implicit theory asserts that some basic elements of creativity are consistently constructed in individuals’ minds despite that different people have diverse views on creativity. Drawing on this assertion, the authors used literature review and in-depth interviews to develop scale items. The scale includes 16 items in three dimensions of divergent thinking (e.g., I can come up with new ideas or solutions to the problems encountered in learning), intelligent application (e.g., I can transform innovative ideas into beneficial applications) and personality characteristics (e.g., I like questioning). Each item in the scale is scored from 1 (strongly disagree) to 5 (completely agree). The scale has good reliability and validity, with internal consistency coefficients of the overall scale and three dimensions range from 0.672–0.867, and the confirmatory factor analysis fitting index being χ^2^/df = 2.34, RMSEA = 0.063, GFI = 0.922, CFI =0.907. In the present study, the internal consistency coefficient was 0.920.

#### 2.2.3. Self-directed learning questionnaire

The self-directed learning questionnaire was used to assess healthcare undergraduates’ SDL. The questionnaire was developed by Song (2020) based on self-regulation theory, including 25 items in four dimensions of meta-cognition ability (e.g., I will adjust my learning plan according to the specific situation during the learning process), learning motivation (e.g., I often choose complex learning tasks), learning strategies (e.g., I often learn new knowledge with questions) and learning grit (e.g., No matter what setbacks I encounter, I will complete the goals as long as I start). Each item was rated by using a five-point Likert scale, with options ranging from 1 (strongly disagree) to 5 (completely agree). The internal consistency coefficients for the overall questionnaire and four dimensions ranged from 0.782 to 0.926 in the present study, and the factor load coefficients were 0.441–0.609, demonstrating its good reliability and validity.

#### 2.2.4. Openness to diversity and challenges scale

The Openness to Diversity and Challenges Scale developed by Pascarella et al. (1996) was used to evaluate healthcare undergraduates’ ODC. It is a unidimensional scale with 8 items that are rated on a five-point Likert scale (1 = strongly disagree; 5 = strongly agree). The sample items included “I enjoy having discussions with people whose ideas and values are different from my own” and “I enjoy taking courses that challenge my beliefs and values.” The scale has been widely used in university settings, with internal consistency coefficient being 0.830 (Alt, 2016; Shim and Perez, 2018). The original English scale was firstly translated to Chinese by the first and second author independently. Thereafter, the third author compared the two Chinese versions to eliminate discrepancies regarding the wording of the statements and finalized the Chinese version of the scale. The Chinese version was back-translated into English by one proficient bilingual academic who was not exposed to the original scale before. The original and back-translated versions were compared by another bilingual academic who reported no meaning difference between the two versions that were highly consistent in content and semantics. In the present study, the Cronbach’s alpha of the Chinese version scale was 0.871.

#### 2.2.5. The Chinese creative self-efficacy scale

The Chinese Creative Self-efficacy Scale was employed to measure students’ CSE, which was developed by Yang (2007) based on Bandura’s Self-efficacy Theory (Bandura, 1977) and creativity components (Guilford and Hoepfner, 1971). The scale consists of 21 items across 4 dimensions of sensitivity (e.g., I can identify defects and areas for improvements in daily necessities), flexibility (e.g., When I find an idea does not work, I can rapidly change my way of thinking to seek other solutions to a problem), ingenuity (e.g., I can put forward new questions from familiar knowledge or phenomena), and fluency (e.g., I can think issues from multiple aspects, various angles, and diverse levels). Each item was rated on a five-point Likert scale (1 = totally impossible; 5 = totally possible). The internal consistency coefficients for the scale and four dimensions were 0.68–0.89, and the factor load coefficients were 0.46–0.66 (Yang, 2007). In the current study, the Cronbach’s alpha of the scale was 0.949.

### 2.3. Data analyses

All statistical analyses were performed using SPSS software version 26.0. Descriptive statistics were used to describe the basic information of participants’ characteristics (e.g., frequency and percentage) and the study variables (e.g., mean and standard deviation). Given that data were collected in a self-reported form in this study, Harman’s single factor test was performed to detect the common method bias effect. Pearson correlations between the study variables were calculated. A hierarchical multiple linear regression analysis was conducted to examine the potential mediating roles of ODS and CSE between SDL and creativity. First, control variables were input in Block 1. Second, SDL was added (Block 2). Third, ODC was added (Block 3). Finally, CSE was added (Block 4). To further analyze the indirect effects of SDL on creativity through ODC and CSE, Structural Equation Modeling was utilized by AMOS 26.0. The bootstrapping method (5,000 resamples) was employed to estimate the 95% bias-corrected confidence interval (BC CI) for the indirect effects of mediators (Preacher and Hayes, 2008). When the 95% confidence interval does not contain zero, the indirect effect is deemed to be significant. A *p* < 0.05 (two tailed) was used to determine statistical significance.

## 3. Results

### 3.1. Participants’ characteristics

The average age of participants was 19.28 ± 1.124 years (age range 17–23), including 256 males (44.5%) and 319 females (55.5%), 172 freshmen (19.9%), 166 sophomores (28.9%), 167 juniors (29.0%), and 70 seniors (12.2%). Among the participants, 202 (35.1%) were the only child in their family and about a half (328, 57.0%) resided in urban area. For academic performance, about one third (195, 33.9%) reported on average compared to their fellow students, 126 students (21.9%) reported below the average, and 254 students (44.2%) reported above the average. Through independent samples *t-*test and one-way analysis of variance (ANOVA), students’ academic performance, education level of father and mother, family income, family residence, were significant factors of creativity (*p* < 0.05). As a result, these factors were entered as control variables in the hierarchical multiple linear regression model, and as covariates in the mediator models. The detailed information is presented in Table 1.

**Table 1:** Characteristics of participants and difference in creativity.

Variables		n (%)	M ± SD	*t/F*	*p*
Gender	Male	256 (44.5)	59.72 ± 11.69	1.625	0.105
	Female	319 (55.4)	58.21 ± 10.50		
Grade	Freshman	172 (29.9)	60.08 ± 9.36	1.802	0.146
	Sophomore	166 (28.9)	59.37 ± 12.06		
	Junior	167 (29.0)	57.84 ± 10.82		
	Senior	70 (12.2)	57.24 ± 12.71		
Academic performance	Blow the average	126 (21.9)	56.65 ± 11.50	7.598	0.001
	On average	195 (33.9)	57.79 ± 10.04		
	Above the average	254 (44.2)	60.82 ± 11.30		
Being a student leader or not	Yes	201 (35.0)	59.21 ± 12.42	0.518	0.605
	No	374 (65.0)	58.70 ± 10.26		
Being the single child or not	Yes	202 (35.1)	59.80 ± 11.54	1.473	0.141
	No	373 (64.8)	58.38 ± 10.77		
Family residence	Urban	328 (57.0)	60.25 ± 11.53	3.454	0.001
	Rural	247 (43.0)	57.06 ± 10.14		
Father’s education level	Primary school or below	93 (16.2)	56.37 ± 9.08	7.765	<0.001
	Junior middle school	208 (36.2)	57.00 ± 11.12		
	High school or equivalent	148 (25.7)	61.33 ± 10.47		
	College and above	126 (21.9)	60.95 ± 12.04		
Mother’s education level	Primary school or below	145 (25.2)	56.75 ± 9.58	5.294	0.001
	Junior middle school	187(32.5)	57.88 ± 11.15		
	High school or equivalent	139 (24.2)	60.55 ± 10.65		
	College and above	104 (18.1)	61.42 ± 12.57		
Family income	Blow the average	200 (34.8)	56.50 ± 11.15	8.032	<0.001
	On average	321 (55.8)	59.87 ± 10.43		
	Above the average	54 (9.4)	61.83 ± 12.82		

### 3.2. Common method deviation test

Harman’s single factor test was applied to assess the potential common method deviation caused by the self-report questionnaire method. The test evinced 9 factors with eigenvalues greater than 1, and the variation explained by the first factor was 35.11%, which was below the threshold value of 40% (Xiong et al., 2012). This demonstrates that the effect of common method deviation would not influence the interpretation of data analysis results.

### 3.3. Bivariate correlations among creativity, SDL, ODC, and CSE

Table 2 shows that creativity, SDL, ODC, and CSE were significantly and positively correlated at the 1% level, suggesting that further mediation effects could be tested. Specifically, creativity was positively and highly correlated with SDL (*r* = 0.721, *p* < 0.001), with CSE (*r* = 0.572, *p* < 0.001) and with ODC (*r* = 0.726, *p* < 0.001), respectively. SDL was positively associated with both CSE (*r* = 0.527, *p* < 0.001) and ODC (*r* = 0.628, *p* < 0.001). CSE was positively related to ODC (*r* = 0.486, *p* < 0.001).

**Table 2:** Mean (M), standard deviations (SD), and correlations between the variables.

Variables	*M*	*SD*	Creativity	SDL	CSE	ODC
Creativity	58.88	11.06	1			
SDL	83.56	10.69	0.721***	1		
CSE	71.91	12.57	0.572***	0.527***	1	
ODC	25.13	4.43	0.726***	0.628***	0.486***	1

SDL, Self-directed learning; ODC, Openness to diversity and challenge, CSE, Creative self-efficacy.

***p < 0.001 (two tailed).

### 3.4. The result of the hierarchical multiple regression

As shown in Table 3, SDL, ODC, and CSE explained 66.2% of the variance in creativity. SDL positively predicted creativity (*β* = 0.706, *p* < 0.001), ODC positively predicted creativity (*β* = 0.451, *p* < 0.001) and CSE played a positive predictive role (*β* = 0.169, *p* < 0.001). When ODC and CSE were added in Step 3 and Step 4 sequentially, the regression coefficient of SDL on creativity decreased from 0.706 to 0.425 and from 0.706 to 0.369, respectively. Likewise, when CSE was added in Step 4, the regression coefficient of ODC on creativity decreased from 0.451 to 0.408. The results suggest that ODC and CSE act as potential mediators between SDL and creativity, and ODC-CSE play a chain mediating role.

**Table 3:** Hierarchical multiple linear regression analysis results.

Variables	Creativity			
	Step 1 (*β*)	Step 2 (*β*)	Step 3 (*β*)	Step 4 (*β*)
Block 1				
Father’s education level	0.080	0.024	0.007	−0.002
Mother’s education level	0.042	0.010	0.007	0.006
Family residence	0.057	0.053	0.053	0.039
Family income	0.089	0.006	0.005	0.001
Academic performance	0.112	0.018	0.024	0.011
Block 2				
Self-directed learning		0.706	0.425	0.369
Block3				
Openness to diversity and challenge			0.451	0.408
Block 4				
Creative self-efficacy				0.169
*R*^2^	0.059	0.525	0.648	0.667
△*R*^2^	0.050	0.520	0.644	0.662

### 3.5. Analyses of direct and indirect effects

**Figure 2:**
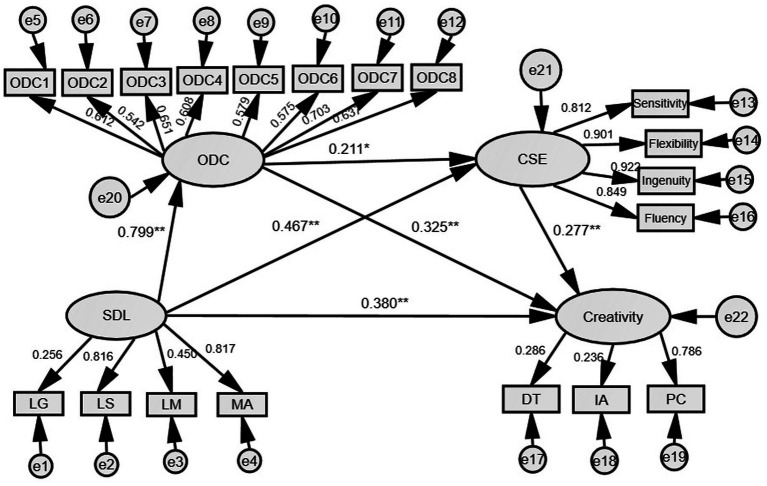
The double chain mediation model of openness to diversity and challenge and creative self-efficacy as mediators of the effect of self-directed learning on creativity. **p < 0.001, *p < 0.05 (two-tailed). SDL, Self-directed learning; ODC, Openness to diversity and challenge; CSE, Creative self-efficacy; LG, Learning grit; LS, Learning strategies; LM, Learning motivation; MA, Meta-cognition ability; DT, Divergent thinking; IA, Intelligent application; PC, Personality characteristics.

The results of bias-corrected percentile bootstrap analysis revealed significant indirect effects of ODC and CSE on the relationship between SDL and creativity (Table 4 and Figure 1). The total effect of SDL on creativity was 0.606 (SE = 0.048, *p* < 0.001, boot 95% BC CI [0.510, 0.695]). The total direct effect and indirect effect of SDL on creativity was 0.282 (SE = 0.118, *p* < 0.001, boot 95% BC CI [0.045, 0.502]), and 0.324 (SE = 0.095, boot 95% BC CI [0.165, 0.543]), respectively. The ration of indirect effect of SDL on creativity to the total effect was 53.47%, indicating that ODC and CSE played partial mediating effects. The mediating effects encompassed three indirect effects. Namely, Path 1: SDL → ODC → creativity (estimated effect = 0.193); Path 2: SDL → CSE → creativity (estimated effect = 0.096); and Path 3: SDL → ODC → CSE → creativity (estimated effect = 0.035). As displayed in Table 4, all the three paths were significant because their 95% CI did not include zero. The mediation effects of the three paths accounted for 59.57, 29.63, and 10.80% of the total indirect effects, respectively. The results verified the hypotheses regarding the direct and indirect effects of SDL on creativity, and the partial mediating effects of ODC and CSE. Figure 2 shows the standardized path coefficients of the proposed serial multiple mediation model, representing the direct path coefficients between the variables.

**Table 4:** Total, direct and indirect effects in the multiple mediator model.

Model	Estimated effect	Boot SE	*p*	Boot LLCI	Boot ULCI	Relative mediation effect
Total effect of SDL on creativity	0.606	0.048	<0.001	0.510	0.695	–
Total direct effect of SDL on creativity	0.282	0.118	<0.001	0.045	0.502	–
Total indirect effect of SDL on creativity	0.324	0.095	<0.001	0.165	0.543	53.47%
Indirect effect 1: SDL → ODC → creativity	0.193	0.088	0.012	0.054	0.401	31.85%
Indirect effect 2: SDL → CSE → creativity	0.096	0.030	0.001	0.048	0.172	15.84%
Indirect effect 3: SDL → ODC → CSE → creativity	0.035	0.018	0.031	0.004	0.076	5.78%

SDL, Self-directed learning; ODC, Openness to diversity and challenge; CSE, Creative self-efficacy. Boot SE, Standard error of indirect effects; Boot LLCI, the lower bound of the 95% confidence interval; Boot ULCI, the upper limit of the 95% confidence interval (Percentile Bootstrap Method with Bias Correction).

## 4. Discussion

Creativity and SDL are critical competences that are worthy to be fostered in educational settings (Morris, 2020), particularly for healthcare students to enable them to tackle challenges in contemporary complex healthcare settings. The present study evinced that SDL, ODC and CSE were all positively correlated with creativity among healthcare undergraduates. In particular, SDL has a significant and positive predictive effect on creativity, with ODC and CSE playing partial and chain mediation effects in the relationship between SDL and creativity. The results suggest that a higher level of SDL was conductive to developing ODC and promoting CSE for healthcare undergraduates, thereby further lead to a greater possibility of higher level of creativity. The findings illustrate the underlying mechanism through which SDL affects creativity.

### 4.1. The direct effect of SDL on creativity

Self-directed learning in medical education was conceptualized as a fundamental attribute of professional identity (Ricotta et al., 2022). SDL represents a pragmatic process of solving or resolving real-world based problems (Morris, 2020), during which individuals may produce creative solution to problems and thus SDL support creative learning outcomes. SDL was viewed as a one of the intrinsic factors influencing creativity (Ma et al., 2018). The present study confirmed the positive link between SDL and creativity, and H1 was validated. Similarly, in an investigation study among first-year undergraduates in Hong Kong, SDL positively influenced cognitive learning outcomes including thinking creatively, analytically and critically, and self-growth outcomes (e.g., ability to have critical self-reflection) (Zhoc et al., 2018), which benefit the enhancement of creativity.

Self-directed learning has been proved to be effective in students’ independent thinking, reflection and creativity development. Based on self-determination theory, SDL offers students more control over their own learning and signifies more autonomous learning strategies (Kemp et al., 2022). In this context, students need to resolve some tasks on their own that were often carried out by the educator in the traditional lecture-formatted educational programs. Through the self-directed learning process, students constructed knowledge through observation, reflection, practice, problem discovery and resolution (Wang and Ji, 2021). Consequently, this process needs students’ active and persistent engagement. In this sense, self-directed learning facilitating creativity partly rests with its link to enhanced cognitive persistence that is one pathway to creativity proposed in the Dual Pathway to Creativity Model (Baas et al., 2013).

Particularly, in an open networked learning environment, students with a higher level of SDL search for useful information and direct their own learning when they develop ideas and connect with others on networks, which provide new opportunities to enhance their learning experiences (Kop and Fournier, 2010). Accordingly, in the process of SDL, students probably apply creative abilities and a flexible mindset to critically analyze resources and filter information. Morris (2020) argued that self-directed learning is underpinned by constructivist epistemology in which individuals may start to view knowledge in a different way in various contexts. Congruently, Alt (2016) discussed that students taking responsibility for their learning are encouraged to become critical thinkers and possess creativity to apply a different approach to deal with problems. The result regarding the positive and predictive role of SDL in creativity in the present study corroborates the findings of previous research.

Given the fact that student-centered medical education are not widely implemented in China (Wang, 2021), the study finding of the direct effect of SDL on creativity suggest that the educators need to recognize the strengths of SDL and create supportive SDL environment where healthcare undergraduates are motivated to be self-directed learners in order to cultivate their creativity. According to the Person Process Context model of SDL, although SDL is primarily a learner-driven process, faculty and peers play a crucial part in guiding and promoting SDL by offering information and feedback to co-construct relevant knowledge (Sawatsky et al., 2018; Morris, 2020). Educational strategies such as placing students in authentic learning situations (van Woezik et al., 2021) and collaborative learning environment in the form of group/team work (Kemp et al., 2022) have been effective to promote SDL in medical education. In addition, both case-based and problem-based learning are inquiry-oriented forms of learning that enable a pragmatic self-directed learning process (Morris, 2020), and thus are confirmed effective in improving problem-solving and self-learning skills in undergraduate medical education (Trullàs et al., 2022). Furthermore, educators need to foster both affective cohesion (e.g., feelings of trust) and behavioral cohesion (e.g., participation) in group activities when design curriculum and/or training program due to the significance of group cohesion in enhancing SDL (Kim and Yang, 2020). These strategies are in line with advocacy of self-determination theory in terms of supporting learners’ sense of autonomy, competence, and relatedness, and hence could be drawn on to cultivate SDL ability for healthcare undergraduates, which in turn benefit the training of creativity.

### 4.2. Indirect effects of SDL on creativity

Notably, the current study uncovered the mechanism of how SDL influenced creativity among healthcare undergraduates. Bootstrapping showed that SDL exerted indirect effects on creativity through ODC, which accounted for 35.89% of the total effect. Namely, ODC mediated the positive effect of SDL on creativity. Previous research demonstrated that undergraduates reported higher levels of ODC in more constructivist learning environments where students actively engaged in SDL (Alt, 2016). Similarly, a previous study reported a significant and positive correlation between learners’ self-directedness and the personality trait of openness (Cazan and Schiopca, 2014). In consistent with the previous research, the present study evinced that SDL was a predictor of ODC and has the potential to enhance ODC.

A qualitative study on medical students, postgraduate medical trainees, and specialists perceived that being open-minded to different perspectives is a vital prerequisite to develop creativity (Ten Haven et al., 2022). Students who are open to diversity and challenge accept and welcome challenges to their beliefs (Bowman, 2014), which may enable new perspectives helping to critically reflect on problems and possible solutions. Openness enables students to challenge group thinking that reinforces critical thinking closely linked with creativity (van Woezik et al., 2021). A study showed that ODC was positively associated with divergent thinking that is a key cognitive process of creativity (Zhu and Doo, 2022). In addition, openness to experience was found to positively correlated with different types of creativity (e.g., scholarly creativity, artistic creativity) among college students (Tidikis and Dunbar, 2019). Based on the Dual Pathway to Creativity Model, ODC influenced creativity mainly through its impact on the cognitive flexibility pathway (Baas et al., 2013). Furthermore, from a neurological perspective, researchers uncovered that openness to experience predicted dopamine effects on divergent thinking (Kackenmester et al., 2019), and was related to creative achievements in the primary sensorimotor brain network (Zhu and Doo, 2022). In fact, openness to experience focuses on differences and thus varies from ODC that highlights both differences and challenges occurring in diverse interactions and experiences (Bowman, 2014). In this sense, the present study extends previous research via substantiating the mediating effect of ODC on the relationship between SDL and creativity.

In view of the mediating role of ODC, more efforts are required by educators to improve learning environments by creating opportunities or motivating students to make connections between their learning and the world, in which they encounter diverse views and perspectives on life and the world (Álvarez-Huerta et al., 2022). Specifically, establishing cooperative learning environment (Alt, 2016) and exposing students to new places and people such as designing study in interdisciplinary teams (Liu, 2022), arranging participation in out-of-class experiences (e.g., clinical placement or rotation in different hospitals) (Ten Haven et al., 2022), and collaborating with industry partners (e.g., health technology companies) (Yuen and Balakrishnan, 2019) could be effective approaches to facilitating ODC of students. Besides, stimulating students to think ‘outside of the box’ by brainstorming in an open-minded form, and inviting them to provide multiple solutions were considered useful techniques to boost creativity (Ten Haven et al., 2022).

Simultaneously, CSE played an independent mediating role, similar to ODC. In other words, SDL was positively associated with CSE that in turn bolstered creativity. A recent qualitative analysis revealed that medical students reported increased belief in themselves and their abilities from SDL experiences (Kemp et al., 2022). The boost of confidence can be attributed to SDL promoting students’ sharing knowledge and resources with peers, as well as critical reflection on their experience. This explains the finding that SDL contributes to increased CSE in the present study. CSE is concerned about ones’ perceptions or beliefs of their creative capability (Tierney and Farmer, 2002), which serves as a driving force to propel individuals to engage in creative activities and persist in these activities (Gong et al., 2019). The predictive role of CSE in creativity has been well documented in literature. For instance, CSE mediated the relationship between critical thinking disposition and scientific creativity (Qiang et al., 2020), between creative mindsets and creative problem solving (Royston and Reiter-Palmon, 2017), and between creative potential and creative achievement (Karwowski, 2016). The present study strengthens the mediating effect of CSE on the relationship between SDL and creativity. Therefore, strategies aimed to improve CSE that targeted the four key sources of CSE (i.e., mastery experiences, vicarious experiences, verbal persuasion, and physiological affective states) (Puente-Diaz, 2016) could be integrated into programs with a focus on fostering creativity among healthcare undergraduates. Drawing on the teaching strategies that a study proved effective to increase CSE among biochemistry undergraduates in UK (Payne and Whitworth, 2022), educators for healthcare undergraduates could harness and implement measures including providing practice sessions with virtual or standardized patients, facilitating discussion between students in interprofessional education course, and dividing challenging tasks (e.g., case scenario) into smaller sub-tasks to raise their CSE.

### 4.3. The chain mediating effects

Distinctively, ODC and CSE played a chain-mediating role in the mechanism of SDL affecting creativity. In other words, SDL first promoted students’ ODC, and then ODC increased CSE, which, in turn, contribute to creativity. The underlying mechanism could be that students with better ability of SDL were more open to diversity and challenge, which raised their confidence in creative activities. Previous studies consistently identified a positive relationship between ODC and creative confidence beliefs (Álvarez-Huerta et al., 2022). ODC empowers students to engage in meaningful educational experiences and quality peer interactions (Bowman, 2014), thereby enhances the faith in their capacity to generate something creative. Additionally, students who are more open to diversity and challenge show more willingness to try new things and consider new ideas (Bowman, 2014), which is an essential prerequisite for creativity. The chain mediation role of ODC-CSE further highlights the value of addressing both ODC and CSE in pertinent programs to maximize the effectiveness of SDL on creativity among healthcare undergraduates.

Overall, SDL exerted both direct and indirect effects on creativity and the two paths had similar weights. The findings shed lights on multiple pathways to enhance creativity in terms of fostering students’ SDL, encouraging ODS and improving CSE. Higher education administrators and practitioners could harness the findings to formulate pertinent strategies to support student creative development for better responding to highly demanding healthcare service. Specifically, the sequential mediating roles of ODC and CSE in the association between SDL and creativity offer a new perspective to promote the development of these skills in higher education.

### 4.4. Limitations and future directions

There are some limitations of the current study that suggest directions for future research. First, the participants were selected using convenience sampling from a large public university and relatively homogeneous, which results in limited generalizability of the findings to all healthcare undergraduates in different institutions in diverse geographic areas. Further studies involving students from other discipline, universities and countries are warranted. Second, all variables in the study were measured using self-reporting scales, which may lead to some potential social desirability response bias when estimating the associations. Application of more objective measures and a longer period of time follow-up would strengthen the findings. In addition, previous research reported that critical thinking disposition (Álvarez-Huerta et al., 2022) and emotional intelligence (Zhoc et al., 2018) influenced creativity, and thus may be the potential covariates exerting impacts on the coefficients in the model in the presented study. Future study could consider these variables to examine their effects by alternative models. Despite the limitations, to our knowledge, this study is one of the first elucidating the associations between SDL, ODC, CSE and creativity of healthcare undergraduates. The present study provides insights into the underlying mechanisms through which SDL influence creativity and highlights the distinct mediating roles of ODC and CSE.

## 5. Conclusion

The study demonstrated that SDL, ODC, CSE and creativity were highly interrelated among healthcare undergraduates. SDL directly and indirectly affected creativity. ODC and CSE played a chain mediating role in the link between SDL and creativity. The findings shed light on multiple pathways to cultivate students’ creativity. Higher education administrators and practitioners create a constructive learning environment and formulate related intervention programs to support student development of SDL ability, increase their ODC and boost their CSE, which together contribute to enhanced creativity.

## Data availability statement

The raw data supporting the conclusions of this article will be made available by the authors, without undue reservation.

## Ethics statement

The studies involving human participants were reviewed and approved by the Ethics Committee of the Medical College of Qingdao University. The patients/participants provided their written informed consent to participate in this study.

## Author contributions

JQ and TL designed the study, analyzed the data, and revised the manuscript. XL, MZ, and KL collected the data. JQ and XL wrote the manuscript. All authors contributed to the article and approved the submitted version.

## Funding

This research was supported by a grant from Qingdao University where the study was conducted as a student innovation project (X2022110650216).

## Conflict of interest

The authors declare that the research was conducted in the absence of any commercial or financial relationships that could be construed as a potential conflict of interest.

## Publisher’s note

All claims expressed in this article are solely those of the authors and do not necessarily represent those of their affiliated organizations, or those of the publisher, the editors and the reviewers. Any product that may be evaluated in this article, or claim that may be made by its manufacturer, is not guaranteed or endorsed by the publisher.
